# Low-level red-light therapy for myopia control in children: A systematic review and meta-analysis

**DOI:** 10.1016/j.clinsp.2024.100375

**Published:** 2024-05-09

**Authors:** Dillan Cunha Amaral, Sávio Batista, Edson dos Santos-Neto, José Eduardo Ferreira Manso, Márcio Penha Morterá Rodrigues, Mário Luiz Ribeiro Monteiro, Milton Ruiz Alves, Ricardo Noguera Louzada

**Affiliations:** a Faculdade de Medicina, Universidade Federal do Rio de Janeiro, Rio de Janeiro, RJ, Brazil; b Division of Ophthalmology and the Laboratory for Investigation in Ophthalmology (LIM-33), Faculty of Medicine, University of São Paulo, São Paulo, SP, Brazil

**Keywords:** Low-level red-light therapy, Myopia, Children, Axial length, Cycloplegic spherical equivalent error, Adverse effects

## Abstract

**Introduction:**

Low-Level Red-Light (LLRL) Therapy is a safe and natural way to promote healing and reduce inflammation in the body. When it comes to treating myopia in children, LLRL therapy is recent, and its efficacy and safety still are not clear.

**Methods:**

A systematic review and meta-analysis of the literature for LLRL was conducted in accordance with the PRISMA guidelines on November 5, 2022. Databases, including PUBMED, Cochrane Library, Web of Science, and Embase were queried. A meta-analysis of random effects was conducted. Inclusion criteria included Randomized Controlled Trials (RCTs) or observational studies where LLRL therapy was used in children (3‒15 years old) with myopia. Exclusion criteria were studies with other ocular abnormalities. Efficacy was evaluated through the mean change in Axial Length (AL) and cycloplegic Spherical Equivalent Error (SER), while safety was evaluated by monitoring adverse effects.

**Results:**

A total of 5 final studies were included (4 RCTs, and 1 observational), in which 685 total patients were analyzed. The mean age was 9.7 ± 0.66 years, with 48,2% female patients. The number of eyes in the LRLL arm is 714 and, in the control, arm is 656. LLRL showed better results in SER and AL mean change (OR = 0.58; 95% CI 0.33 to 0.83; p < 0.00001, and MD -0.33; 95% CI -0.52 to -0.13; p = 0.001, respectively), in comparison to the control group. There was no significant difference in adverse effects between groups (MD = 5.76; 95% CI 0.66 to 50.14; p = 0.11).

**Conclusion:**

LLRL therapy is a non-invasive, effective, and safe short-term treatment option; however, long-term evaluation, particularly in comparison to other therapies, requires additional investigation.

## Highlights

• There is a global increase in childhood myopia and current treatments are limited.
• LLRL non-invasive therapy shows promise in myopia control.
• LLRL is promising and requires further studies for standardization and safety.

## Introduction

Myopia is a refractive disorder of the eye that is increasingly prevalent.^1^ It is estimated that in 2000, 1.4 billion people were myopic, and it is predicted that by 2050 the number will reach 4.8 billion.^2^ Children with early diagnosis of myopia are the major group risk because they will have a longer duration of the disease and higher myopia progression with an increased risk of developing high myopia plus other complications.^2^ In the general population, myopia prevalence remains higher in Asia (60%) compared with Europe (40%) using cycloplegic refraction examinations. Otherwise, a low prevalence of under 10% was described in African and South American children.^2^ Furthermore, in recent studies, risk factors for myopia in schoolchildren are low outdoor time, dim light exposure, the use of LED lamps for homework, low sleeping hours, a reading distance of less than 25 cm, and living in an urban environment.^3^ Thus, the disorder has significant public healthcare implications worldwide and represents a significant societal and economic responsibility to healthcare systems globally.^1^^,^^4^ Socioeconomically, refractive errors like myopia, particularly if uncorrected, can affect school performance, limit employability and impair quality of life.^2^^,^^5^

The treatment of myopia has some options.^6^ Atropine, ortho-K contact lenses, and soft bifocal contact lenses have been shown to be the most effective ones.^3^ However, ortho-K contact lenses and soft bifocal contact lenses are associated with a major risk of sight-threatening infectious keratitis.^7^ Atropine in higher therapeutic doses has limited practical use because of pupil dilatation, loss of accommodation, and near vision blur.^8^ However, patient compliance is an issue, with high dropout rates reported in some trials.^9^ Therefore, these therapies are limited in some variables like treatment compliance, potential side effects, and lack of long-term data.

Low-Level Red-Light (LLRL) therapy is another safe and natural way to promote healing and reduce inflammation in the body.^10^ In medicine, LLRL therapy is used to penetrate deep into the body and stimulate natural healing processes. This type of therapy is non-invasive, painless, and has been proven to be beneficial in treating a wide range of conditions.^11^ When it comes to treating myopia in children, LLRL therapy is recent and its efficacy and safety still are not entirely clear. In light of this controversy, the authors performed a meta-analysis evaluating the efficacy and safety of LLRL compared to control in children with myopia.

## Material and methods

### Search strategy and data extraction

A systematic review and meta-analysis of the literature for low-level red-light therapy was conducted in accordance with the PRISMA guidelines.^12^ This study was registered in the International Prospective Register of Systematic Reviews (PROSPERO; CRD42024504745). The terms: (myopia OR “short-sightedness” OR nearsightedness) AND (“low-level red-light therapy” OR LLRL OR “low-level laser light therapy” OR “low-power laser therapy” OR “non-thermal LED light” OR “soft laser therapy” OR “cold laser therapy” OR “biostimulation” OR “photonic stimulation” OR “photobiomodulation” OR “phototherapy” OR “red light therapy” OR “low-level red light”) was used for the search. The search terms were queried using Pubmed, Embase, Cochrane, and Web of Science databases. The references from all included studies, previous systematic reviews, and meta-analyses were also searched manually for any additional studies. Two authors (D.A. and S.B.) independently extracted the data following predefined search criteria and quality assessment.

### Eligibility criteria

Inclusion in this meta-analysis was restricted to studies that met all the following eligibility criteria:

(1) randomized trials or nonrandomized cohorts;
(2) comparing LLRL therapy to control;
(3) enrolling myopic children 3‒15 years old. In addition, studies were included only if they reported any of the clinical outcomes of interest.

The authors excluded studies with (1) no control group; and (2) patients without myopia or who are not in the desired age range.

### Endpoints

Efficacy outcomes included Axial Length (AL) and cycloplegic Spherical Equivalent Error (SER). Adverse effects were the safety outcome of interest.

### Statistical analysis

This systematic review and meta-analysis was performed in accordance with the Cochrane Collaboration and the Preferred Reporting Items for Systematic Reviews and Meta-Analysis (PRISMA) statement guidelines.^13^ Odds ratios (OR) with 95% Confidence Intervals were used to compare treatment effects for categorical endpoints. Continuous outcomes were compared with Mean Differences (MD). Cochran Q test and I^2^ statistics were used to assess for heterogeneity; p-values inferior to 0.10 and I^2^ > 25% were considered significant for heterogeneity. The authors used a fixed-effect model for outcomes with low heterogeneity (I^2^ < 25%) and a random-effect model was used for outcomes with high heterogeneity (I^2^ > 25%). Publication bias was investigated by funnel-plot analysis.^14^ Review Manager 5.3 (Cochrane Centre, The Cochrane Collaboration, Denmark) was used for statistical analysis.^15^

## Results

### Study selection and characteristics

**Figure 1:**
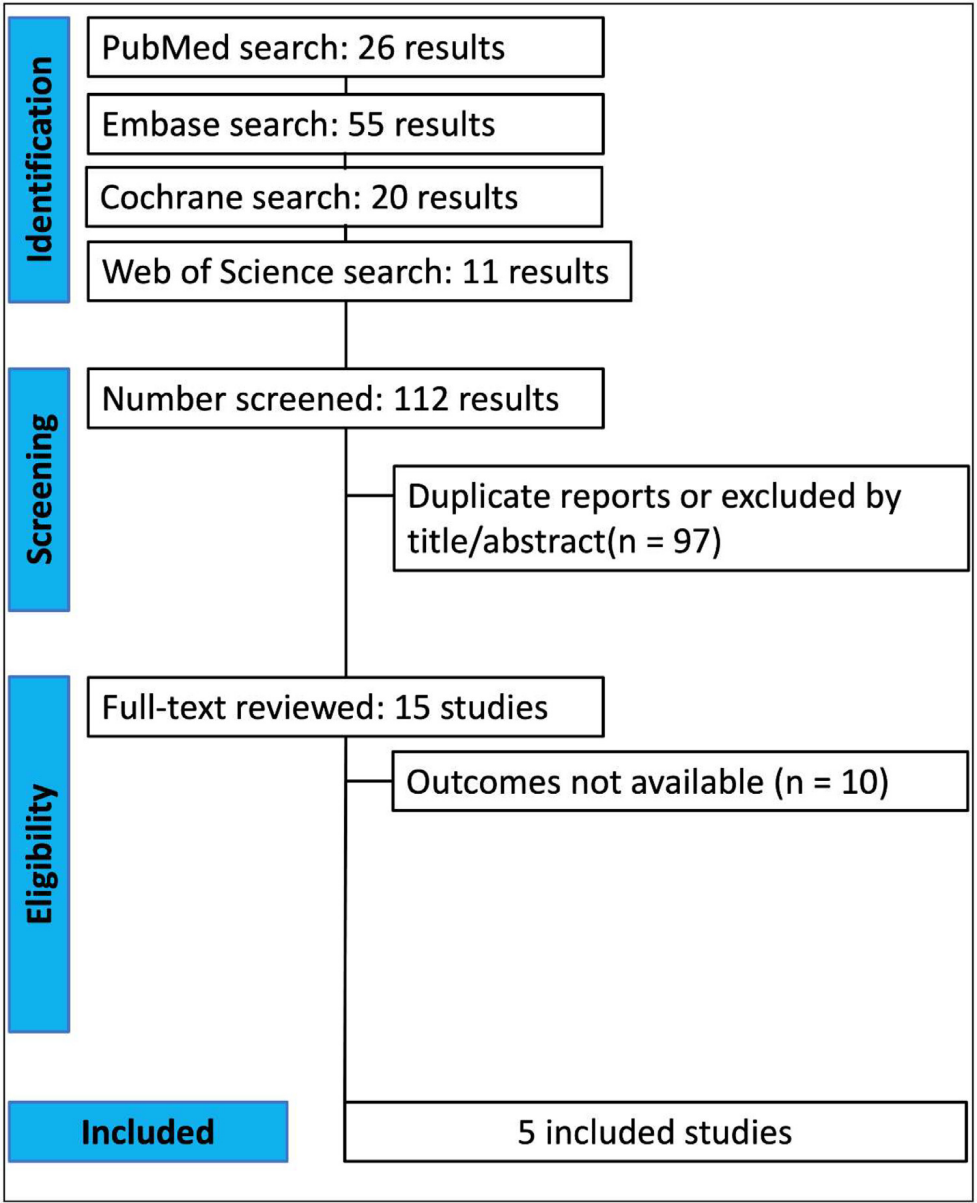
PRISMA flow diagram of study screening and selection.

As detailed in Figure 1, the authors found 112 articles, with 26 in PubMed, 55 in Embase, 11 in Web of Science, and 20 in Cochrane databases. Of these, 34 were removed as duplicates. After the removal of duplicate records and ineligible studies, 15 remained and were fully reviewed based on inclusion criteria. Next, 9 articles were excluded as per the exclusion criteria, and 1 was during the data extraction. Finally, 5 studies were included in this review, 4 Randomized Controlled Trials (RCTs)^16^, ^17^, ^18^, ^19^ and 1 non-randomized cohort.^20^

### Baseline characteristics

A total of 685 patients were analyzed in the present study. The mean age was 9.7±0.66 years, with 48.2% female patients. The number of eyes in the LRLL arm is 714 and in the control, arm is 656. Study characteristics are reported in Table 1.

**Table 1:** Baseline Characteristics of included studies.

**Study**	**Design**	**Follow-up**	**LLRL group**				**Control Group**			
			**M/F ratio**	**Mean age**	**Mean SER**	**Mean AL**	**M/F ratio**	**Mean age**	**Mean SER**	**Mean AL**
Jiang et. al, 2022	RCT	12 mo	57/62	10.4 ± NA	-2.49 ± 0.92	24.54 ± 0.67	73/72	10.5 ± NA	-2.67 ± 1.06	24.62 ± 0.86
Dong et. al, 2022	RCT	6 mo	26/30	10.3 ± 2.07	-3.13 ± 1.91	24.7 ± 1.04	30/26	9.86 ± 1.41	-2.82 ± 1.86	24.6 ± 0.96
Zhou et. al, 2021	OB	6 mo	56/49	9.19 ± 2.40	-3.09 ± 1.74	24.76 ± 1.28	30/26	8.62 ± 2.45	-3.04 ± 1.66	24.77 ± 1.35
Chen Y et. al, 2022	RCT	12 mo	14/17	9.78 ± 1.58	-2.60 ± 1.17	24.48 ± 0.79	17/14	10.31 ± 1.90	-2.59 ± 1.24	24.67 ± 0.98
Chen H et. al, 2022	RCT	12 mo	27/19	9.00 ± 1.90	-2.54 ± 1.04	24.62 ± 0.97	25/15	8.98 ± 1.92	-2.29 ± 0.77	24.57 ± 0.76

NA, Not Applicable; AL, Axial Length; SER, Spherical Equivalent Error; OB, Observational; RCT, Randomized Controlled Trial; Mo, Months; M, Male; F, Female.

### Pooled analysis of all studies

**Figure 2:**
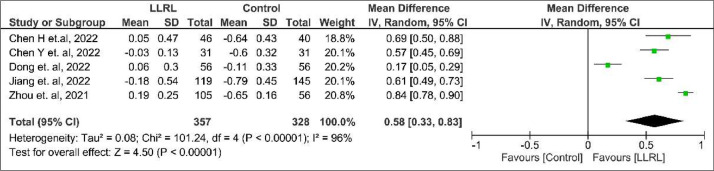
Spherical equivalent error mean change forest plot.

**Figure 3:**
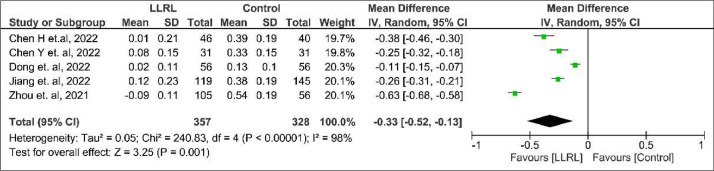
Axial length mean change forest plot.

In comparison to the control group, those receiving LLRL there was a better result towards decreased SER mean change (MD = 0.58; 95% CI 0.33 to 0.83; p < 0.00001; I² = 96%; Fig. 2) and AL mean change (MD = -0.33; 95% CI -0.52 to -0.13; p = 0.001; I² = 98%; Fig. 3).

**Figure 4:**
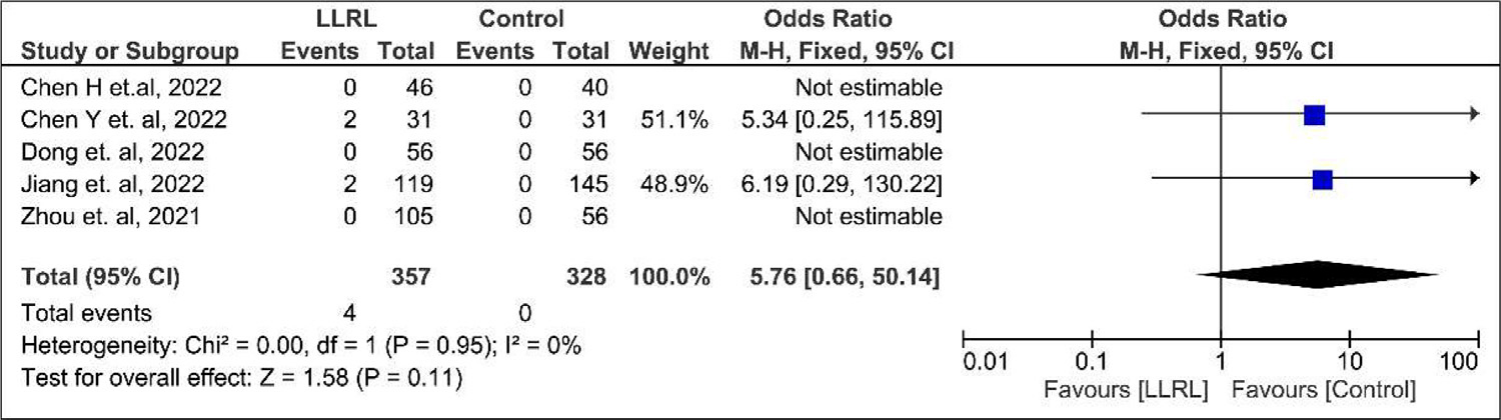
Adverse effects forest plot.

In total, 4 adverse events (2 dizziness and 2 photophobia) occurred in 685 patients. Nevertheless, there was no significant difference in adverse effects between groups (OR = 5.76; 95% CI 0.66 to 50.14; p = 0.11; I² = 0%; Fig. 4).

**Figure 5:**
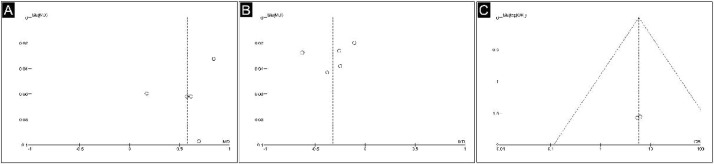
Funnel plot analysis. (A) Spherical equivalent error. (B) Axial length. (C) Adverse effects.

The funnel plot of the included studies appeared relatively symmetrical (Fig. 5), suggesting a low likelihood of publication bias. However, it is important to consider that the accuracy of funnel plots is limited when there are fewer than 10 studies present.^21^

## Discussion

In this systematic review and meta-analysis of 5 studies and 685 patients, the authors compared LLRL to control for the myopia treatment. The main findings with LLRL include better results in SER and AL mean change compared to controlled groups in the pooled analysis of RCT and observational data; and the absence of significant difference in adverse effects between LLRL and control groups.

The efficacy of LLRL has been shown in other previous studies presented in the literature.^16^^,^^22^ Although the comparison with other methods like Atropine and Orthokeratology is difficult because of the study design differences, the low-level red light therapy efficacy results have been reported with competitive values. The notion that LLRL is an effective treatment for myopia is reinforced by the continuity of promising studies.^18^ Thus, the present data helps to confirm the results from previous studies since there is no published meta-analysis on this topic showing the LLRL better results in decreased SER and AL mean change.

The safety of low red light therapy was tested initially by irradiating shaved murine skin.^9^ The research found an unexpected acceleration in hair regrowth and no evidence of neoplastic changes. Thereon, other research performs experimental work to discover the physiological mechanisms of this therapy. Thus, the enhances the metabolic activity of the cell, expression of genes associated with tissue regeneration and repair, and regenerative effort by immune modulation takes place in the photothermal and photoacoustic effects.^9^ In animals and humans, no have been reported adverse side effects in LLRL. Thus, this data helps to confirm the absence of significant differences in adverse effects between LLRL and control groups. So, LLRL is a safe and possibly beneficial approach, based on scientific mechanisms with neurotherapeutic promise for a wide range of ophthalmological and other conditions.^11^ However, further research is needed to understand its long-term safety.

A recent study found that three minutes of uninterrupted exposure to LLRL therapy may reach or exceed the maximum allowable levels for both thermal and photochemical exposure. This poses a potential risk of causing damage to the retina through both thermal and photochemical mechanisms.^23^ As a result, clinicians are advised to exercise caution when using LLRL therapy for treating myopia in children, pending the confirmation of safety standards.

This study has important limitations. First, 1 of the 5 studies were not randomized. Importantly, significant variability in the duration of LLRL and follow-up time between studies was also noted. Substantial variability in the definition of adverse events was also noted between studies. Finally, there was also significant heterogeneity in SER and AL mean change outcomes.

## Conclusion

This systematic review and meta-analysis compared LLRL to control of 685 in myopic children's patients. With a varying duration of LLRL and follow-up time, LLRL appeared to be associated with better results in SER and AL mean change compared to control groups and with no significant difference in adverse effects between LLRL and control groups. Thus, LLRL therapy is a non-invasive, effective, and safe short-term treatment option; however, long-term evaluation, particularly in comparison to other therapies and confirmation of safety standards, requires additional investigation. Larger studies could help confirm and further expand knowledge of the differences between LLRL and other therapies.

## Authors’ contributions

All authors made substantial contributions to conception and design, acquisition of data, or analysis and interpretation of data; took part in drafting the article or revising it critically for important intellectual content; gave final approval of the version to be published; have agreed on the journal to which the article has been submitted; and agree to be accountable for all aspects of the work.

## Funding

This research did not receive any specific grant from funding agencies in the public, commercial, or not-for-profit sectors.

## Declaration of competing interest

All authors report no relationships that could be construed as a conflict of interest. All authors take responsibility for all aspects of the reliability and freedom from bias of the data presented and their discussed interpretation.
